# Understanding Anthracycline Cardiotoxicity From Mitochondrial Aspect

**DOI:** 10.3389/fphar.2022.811406

**Published:** 2022-02-08

**Authors:** Junqi Huang, Rundong Wu, Linyi Chen, Ziqiang Yang, Daoguang Yan, Mingchuan Li

**Affiliations:** ^1^ Key Laboratory for Regenerative Medicine, Ministry of Education, College of Life Science and Technology, Jinan University, Guangzhou, China; ^2^ Department of Biology, College of Life Science and Technology, Jinan University, Guangzhou, China

**Keywords:** anthracycline, cardiotoxicity, mitochondria, ROS, ferroptosis, mitophagy, metabolism

## Abstract

Anthracyclines, such as doxorubicin, represent one group of chemotherapy drugs with the most cardiotoxicity. Despite that anthracyclines are capable of treating assorted solid tumors and hematological malignancies, the side effect of inducing cardiac dysfunction has hampered their clinical use. Currently, the mechanism underlying anthracycline cardiotoxicity remains obscure. Increasing evidence points to mitochondria, the energy factory of cardiomyocytes, as a major target of anthracyclines. In this review, we will summarize recent findings about mitochondrial mechanism during anthracycline cardiotoxicity. In particular, we will focus on the following aspects: 1) the traditional view about anthracycline-induced reactive oxygen species (ROS), which is produced by mitochondria, but in turn causes mitochondrial injury. 2) Mitochondrial iron-overload and ferroptosis during anthracycline cardiotoxicity. 3) Autophagy, mitophagy and mitochondrial dynamics during anthracycline cardiotoxicity. 4) Anthracycline-induced disruption of cardiac metabolism.

## Introduction

Anthracyclines, including doxorubicin, daunorubicin, epirubicin and idarubicin, are a family of antibiotics that are broadly used to treat solid tumors (ovary, breast, stomach, brain, and gastrointestinal tumors) and hematological malignancies (lymphoma and pediatric leukemia) (National Cancer Institute, 2020). Anthracyclines can be used alone or combined with other anti-cancer regimens, such as radiation therapy or monoclonal antibodies (Christodoulou et al., 2009). However, cardiovascular diseases arise as a leading cause of morbidity and mortality among cancer survivors receiving anthracycline therapy, largely limiting the clinical application of these drugs (Zamorano et al., 2016).

At present, there is no standard guideline to prevent anthracycline-associated cardiotoxicity. This is mainly because our understanding of the molecular mechanisms underlying anthracycline cardiotoxicity is still limited. It is currently known that anthracyclines primarily bind to topoisomerase 2 (TOP2) and induce DNA double-strand breaks in cancer cells (Tewey et al., 1984). Similarly in the heart, topoisomerase 2ß (TOP2ß) acts as the main target of anthracyclines in cardiomyocytes (Lyu et al., 2007; Zhang et al., 2012). Formation of ROS due to the redox cycling of anthracyclines is considered as another key mechanism that leads to oxidative stress damage in cardiomyocytes (Simůnek et al., 2009). Beside these, other mechanisms include anthracycline-induced disruption of iron metabolism, insulin resistance and inflammation (Ghigo et al., 2016). More recently, disturbance of cardiac autophagy, especially mitochondrial autophagy (mitophagy), emerges as a newly recognized reason underlying anthracycline cardiotoxicity [reviewed in ref (Li M. et al., 2020)]. Anthracycline cardiotoxicity is likely to be multifactorial and complex [reviewed in refs (Vejpongsa and Yeh, 2014; Ghigo et al., 2016; Sala et al., 2020)]. Nevertheless, distinct mechanisms converge on anthracycline-induced mitochondrial dysfunction as a central event. In the heart, mitochondria are important organelles, which occupy around 30% of the total cardiomyocyte volume and supply 90% energy through oxidative phosphorylation (OXPHOS) for the cardiac biological processes (Piquereau et al., 2013). Thus, deciphering mitochondrial alterations at the molecular and functional levels is critical for anthracycline therapy**.** In this review, we will summarize the recent findings about anthracycline-induced cardiotoxicity, especially about mitochondria-related ROS production, iron-overload and ferroptosis, mitophagy and mitochondrial dynamics disruption, as well as cardiac metabolism alteration after mitochondrial dysfunction.

## Anthracycline Cardiotoxicity: Phenomenon and Current Treatment

Anthracycline treatment results in acute, early and chronic cardiotoxicity. The acute side effects include supraventricular arrhythmia, transient left ventricle dysfunction and electrocardiographic changes, which occur immediately following treatment and are reversible after discontinuation of the therapeutic regimen. However, irreversible cardiac dysfunction may develop early after treatment completion, commonly within 1 year, or manifest several years after treatment (median = 7 years). Irreversible cardiac dysfunction may eventually lead to heart failure and lower the life quality of cancer survivors (Zamorano et al., 2016). For example, breast carcinoma and small cell lung carcinoma patients receiving doxorubicin treatment showed a 5% incidence of congestive heart failure at a cumulative dose of 400 mg/m^2^. This incidence rate rapidly increased to 48% when 700 mg/m^2^ was reached (Swain et al., 2003). Thus, early detection of cardiac dysfunction is important to prevent anthracycline cardiotoxicity. If anthracycline-induced cardiac dysfunction is identified early and interfered with heart failure medications, patients recover easily. In contrast, heart failure is difficult to treat if detected late after anthracycline therapy (Cardinale et al., 2010, 2015).

Phenomenon of chronic anthracycline cardiotoxicity includes enlargement of all chambers and thinning of the ventricular walls, which are classic appearances of a dilated heart, accompanied by continuous progressive decline of fractional shortening and ejection fraction (Vejpongsa and Yeh, 2014). Some studies also reported a decrease of left ventricle mass after anthracycline therapy (Jordan et al., 2018). Morphologically, clinical observations and animal studies both discovered myofibrillar loss, cardiomyocyte atrophy and cellular microvacuolization in anthracycline-treated hearts (Li et al., 2016, 2018). This may explain why chronic anthracycline cardiotoxicity is frequently irreversible. Cardiac fibrosis and inflammation were reported in some cases, although not representative enough (Zhao et al., 2010; Wang et al., 2016).

Strategies of preventing anthracycline-induced cardiac dysfunction include reduction of cumulative dose, liposome-based delivery, continuous infusions or use of less toxic analogues (such as epirubicin) (Vejpongsa and Yeh, 2014; Zamorano et al., 2016). At present, dexrazoxane, an iron chelator, is the only cardioprotective drug approved by Food and Drug Administration (FDA) and European Medicines Agent (EMA) to reduce anthracycline cardiotoxicity. Nevertheless, dexrazoxane was initially restricted to adult metastatic breast cancer patients receiving high cumulative dose of anthracyclines (European Medicines Agency, 2018a). Meanwhile, traditional cardiac medications, including angiotensin converting enzyme inhibitors (ACE inhibitors), angiotensin II receptor blockers (ARBs) and ß-adrenergic receptor antagonists (ß-blockers), are also tested in different studies to prevent anthracycline-associated cardiac side effects. Nevertheless, the results remain controversial, as some studies found a significant improvement of cardiac function by carvedilol (Kalay et al., 2006), nebivolol (Kaya et al., 2013) or carvedilol + enalapril (Bosch et al., 2013), whereas others reported no difference among placebo, metoprolol and enalapril groups (Georgakopoulos et al., 2010). More evidence is needed to clarify this point.

## Anthracycline-Induced Mitochondrial ROS Production

**FIGURE 1:**
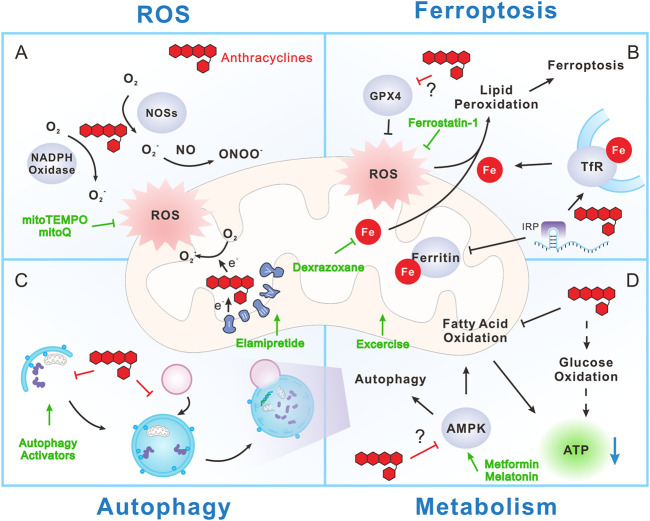
Mitochondrial mechanism during anthracycline cardiotoxicity. **(A)** Anthracyclines promote reactive oxygen species (ROS) production through directly interfering with NADPH oxidase, nitric oxide synthases (NOSs) and mitochondrial electron transport chain (ETC). Mitochondria are the major producers of ROS, but in turn are injured by ROS. **(B)** Anthracyclines can disrupt iron metabolism by interacting with iron regulatory protein (IRP), resulting in promotion of transferrin receptor (TfR) expression and inhibition of ferritin expression. As a result, iron uptake is increased and iron storage is decreased, ultimately leading to free iron overload, especially in mitochondria. ROS can induce lipid peroxidation and consequent ferroptosis in an iron-dependent manner. Notably, anthracyclines inhibit Glutathione peroxidase 4 (GPX4), a phospholipid hydroperoxidase that inhibits lipid peroxidation, thus exacerbate ferroptosis. **(C)** Anthracyclines may disrupt autophagy and mitochondrial autophagy (mitophagy) through inhibition of autophagy initiation and blocking the fusion between autophagosome and lysosome. This prevents the efficient clearance of damaged cellular components including mitochondria and worsens anthracycline cardiotoxicity. However, whether autophagy is protective or detrimental during anthracycline cardiotoxicity is still controversial. **(D)** Anthracyclines largely reduce the utilization of fatty acid while transiently increase glucose oxidation (dashed arrow). The reprogram of fuel substrate utilization does not improve energy supply, but together with mitochondrial dysfunction, eventually lead to ATP reduction and energetic failure. AMPK is the main energy and nutrient sensor that promotes ATP production by activating anabolic processes, such as fatty acid oxidation and autophagy. Notably, AMPK is inhibited by anthracyclines with still unclear mechanism, which further exacerbates anthracycline-induced energetic failure. Strategies aiming at targeting mitochondrial features to reduce anthracycline cardiotoxicity include (green symbols): 1) Mitochondrial specific antioxidants, like mitoTEMPO and MitoQ. 2) Iron chelators, such as dexrazoxane. 3) Ferroptosis inhibitors, like ferrostatin-1. 4) Autophagy activators, such as PI3K/Akt/mTOR inhibitors and AMPK activators (metformin and melatonin). 5) Energetic stimulators, like elamipretide and aerobic exercise.

The most widely accepted mechanism for anthracycline cardiotoxicity is the ability of these drugs to generate excessive reactive oxygen species (ROS) (Figure 1A). The special chemical structures of anthracyclines decide that they can be reduced to a semiquinone form. Inside the cells, this process is catalyzed by nicotinamide adenine dinucleotide phosphate (NADPH) oxidase and nitric oxide synthases (NOSs) in the cytoplasm [reviewed in refs (Rochette et al., 2015; Ghigo et al., 2016)], as well as by mitochondrial electron transport chain (ETC). All these components can transfer electrons to doxorubicin (DOX), for example, to form semiquinone doxorubicin (SQ-DOX). SQ-DOX is an unstable metabolite that could be oxidized by oxygen within mitochondria, accompanied by the release of ROS. This is further worsened by the fact that anthracyclines have high affinity to cardiolipin (Parker et al., 2001), a phospholipid exclusively localized at the inner mitochondrial membrane. Thus anthracyclines preferentially accumulate in mitochondria (Ichikawa et al., 2014). Excess ROS production can induce different types of cellular injury and ultimately lead to cell death. Considering the fact that mitochondria are extremely rich in cardiomyocytes and that the heart has lower levels of antioxidant enzymes, such as catalase and superoxide dismutase (SOD), compared to other organs (Li X. et al., 2020), it is reasonable that the heart is more susceptible to anthracycline-induced ROS generation.

Mitochondrial respiration supplies ATP through electron transfer and the accompanied proton gradient. During this process, ROS are natural by-products under physiological conditions, but may be excessively produced under pathological situations, such as anthracycline stress. Mitochondrial ROS are primarily generated by the ETC complexes, including complex I, II, and III [reviewed in ref (Forrester et al., 2018)]. Complex I (also known as NADH dehydrogenase) is the major site wherein anthracyclines interrupt mitochondrial ROS production. Complex I catalyzes the production of NAD^+^ from NADH and extracts 2 electrons to reduce FMN (flavin mononucleotide), the central unit of complex I. O_2_^−^ is produced when O_2_ reacts with reduced FMN (Forrester et al., 2018). Early studies demonstrated that anthracyclines can facilitate O_2_^−^ generation by extracting electron from complex I to form semiquinone anthracycline. The oxidation of semiquinone anthracycline by O_2_ promotes O_2_^−^ generation (Davies and Doroshow, 1986; Marcillat et al., 1989). In addition, the extraction of electrons by anthracyclines hinders electron flow within ETC and, as a result, reduces ATP production. Later studies further revealed that the activities of complex II and III are also rapidly inhibited by anthracyclines, which do not contribute to ROS generation, suggesting that anthracyclines inactivate cardiac ETC through both oxidative and non-oxidative mechanisms (Marcillat et al., 1989; Pointon et al., 2010).

In summary, anthracycline-induced ROS production is pivotal factor that contribute to cardiomyocyte injury. It is worthy to note that ROS itself could boost secondary ROS generation, a process named ROS-induced ROS release (RIRR) (Zorov et al., 2014). RIRR hypothesis suggests that high level of ROS will cause irreversible opening of mitochondrial permeability transition pore (mPTP), leading to mitochondria damage and consequent ROS release from mitochondria to cytosol. The increased cytosolic ROS is rapidly absorbed by the adjacent normal mitochondria, thereby cascade amplification and release of ROS is achieved, ultimately triggering cell apoptosis (Zorov et al., 2014). In fact, a study in anthracycline-induced endotheliotoxicity models demonstrated that a stable level of cytosolic ROS and rapid rise of mitochondrial ROS were observed in the early phase (4–8 h) after doxorubicin treatment, while a sudden 15-fold increase of cytosolic ROS occurs 16 h after doxorubicin exposure in human umbilical vein endothelial cells (HUVECs), suggesting that RIRR could contribute to the final ROS burst by anthracyclines (He et al., 2020). As anthracycline-induced cardiomyocyte mPTP opening and mitochondrial membrane potential loss are consistently observed in a large number of studies (He et al., 2018; Villa et al., 2021), it is supposed that RIRR also contributes to anthracycline cardiotoxicity, which awaits direct experimental confirmation.

## Mitochondrial Iron-Overload and Ferroptosis During Anthracycline Cardiotoxicity

Disruption of iron homeostasis is another key reason for anthracycline cardiotoxicity. Since their discovery, anthracyclines are found capable of chelating free iron vigorously to form iron-anthracycline complexes, which further react with oxygen and promote ROS production (Gutteridge, 1984). Early studies demonstrated that animals fed with iron-rich food are more susceptible to doxorubicin-induced weight lost and cardiomyocyte apoptosis, suggesting a pivotal role of iron metabolism during anthracycline cardiotoxicity (Panjrath et al., 2007). Since anthracyclines boost ROS production, the iron-anthracycline complexes-related free radicals were initially considered as a contributor to anthracycline cardiotoxicity. Nevertheless, many antioxidant strategies failed to protect against anthracycline-induced myocardium injury in animal studies (Mukhopadhyay et al., 2009) and clinical settings (van Dalen et al., 2011). Thus, iron-anthracycline complexes and the related ROS production may have only limited effect in anthracycline cardiotoxicity. Instead, increasing evidence has supported the view that anthracyclines may directly interrupt iron metabolism, resulting in iron-overload and the consequent ferroptosis (Figure 1B).

### Anthracyclines Interrupt Iron Metabolism

It is progressively clear that free iron accumulates in cardiomyocytes during anthracycline treatment, resulting in iron-overload that could be highly toxic (Ichikawa et al., 2014). Anthracycline-induced interruption of iron metabolism is a complex and multi-target process. Primarily, anthracyclines can interfere with the key iron-binding and -transporting proteins. For example, doxorubicinol, a doxorubicin metabolite, can sequester the Fe-S cluster of iron regulatory protein 1 (IRP-1), which serves as an important feedback regulator that strictly controls the expression of iron metabolism proteins (Minotti et al., 1998, 2001). Iron-doxorubicin complexes further reduce the available free iron pool, leading to a switch of IRP-1 to an iron-free state. Iron-free IRP-1 can bind to the iron-responsive elements (IREs) on the mRNA of iron metabolism-related proteins, such as transferrin receptor (TfR) and ferritin, and regulates their expression (Rouault, 2006). Upregulation of TfR and subsequently iron uptake are detected in doxorubicin-treated cells, whereby anti-TfR antibody effectively reduces doxorubicin-promoted iron uptake and cell death (Kotamraju et al., 2002). Beside disrupting IRP-1, doxorubicin can directly interact with the IREs of ferritin heavy and light chains (Canzoneri and Oyelere, 2008). The combinatorial altered expressions of TfR and ferritin lead to enhancement of iron uptake, inhibition of iron sequestration and ultimately iron-overload in cardiomyocytes. Moreover, deficiency of human hemochromatosis protein (also known as the HFE protein), which competes the interaction between TfR and transferrin, also leads to iron-overload and exacerbates doxorubicin-induced mitochondrial damage and mortality (Miranda et al., 2003). Notably, anthracycline-induced increment of iron contents preferentially accumulates in special subcellular compartments, especially the cardiac mitochondria. This may be due to the fact that mitochondria are the major cellular cites of iron utilization. Iron is required for the synthesis of heme and Fe-S clusters that are essential cofactors of enzymes involved in the tricarboxylic acid (TCA) cycle and the respiratory chain (Rizzollo et al., 2021). Accordingly, hearts from patients with anthracycline-related cardiomyopathy displayed higher iron levels in mitochondria, compared to those from patients with normal cardiac function or other types of heart failure (Ichikawa et al., 2014). Within the matrix, mitochondrial ferritin (FtMt) serves as an iron storage protein in mitochondria to avoid oxidative stress-related damage induced by high iron content. Correspondingly, FtMt deficient mice (FtMt^−/−^) are more susceptible to anthracycine-induced mitochondrial damage, heart remodeling and mortality (Maccarinelli et al., 2014). In contrast, overexpression of mitochondrial iron exporters, such as ABC protein B8 (ABCB8), reduces mitochondrial free iron and protects the heart against anthracycline-induced cardiomyopathy (Ichikawa et al., 2014). Thus, anthracycline-induced disruption of iron metabolism eventually results in labile iron accumulation in mitochondria and finally cardiotoxicity.

### Anthracyclines Induce Ferroptosis in Cardiomyocytes

A long-debated issue about anthracycline cardiotoxicity is which type of programmed cell death contributes mostly to the loss of terminally differentiated cardiomyocytes. It was initially reported that anthracyclines induce DNA damage and apoptosis (Arola et al., 2000). Nevertheless, later evidence suggested that anthracyclines trigger various types of programmed cell death, including necrosis (Zhang et al., 2016), pyroptosis (Wang et al., 2017), and autophagy (Lu et al., 2009). Thereinto, apoptosis, necrosis and pyroptosis are considered detrimental, while autophagy could be cardiac protective (Li et al., 2016, 2018). Intriguingly, disturbance of iron metabolism may eventually lead to another iron-dependent programmed cell death, ferroptosis. In fact, recent studies have highlighted the dominant role of ferroptosis in anthracycline cardiotoxicity (Fang et al., 2019; Tadokoro et al., 2020).

Ferroptosis is a recently identified form of programmed cell death that involves iron- and ROS-dependent damage to membrane lipids. It is characterized by the existence of small mitochondria with condensed mitochondrial membrane, pruning of mitochondria crista, as well as outer mitochondrial membrane rupture (Xie et al., 2016). Ferroptosis is genetically and biochemically distinct from other forms of programmed cell death, like apoptosis, necrosis, and autophagy. It can be triggered by activation of mitochondrial voltage-dependent anion channels, mitogen-activated protein kinases or over-activated endoplasmic reticulum stress. ROS production stimulated by these stresses directly induces peroxidation of phospholipids with polyunsaturated fatty acid (PUFA) chain in an iron-dependent manner, ultimately resulting in ferroptotic cell death. The cystine/glutamate antiporter system Xc^−^ maintains redox homeostasis by importing cystine to produce cysteine, which is used to synthesize the major antioxidant glutathione (GSH), thus inhibiting ferroptosis. In addition, glutathione peroxidase 4 (GPX4), nuclear factor erythroid 2-related factor 2 (NRF2) and heat shock protein beta-1 (HSPB1, also known as HSP27) negatively inhibit ferroptosis by limiting ROS production and reducing cellular iron uptake. In contrast, p53 inhibits SLC7A11, a key component of system Xc^−^, while NADPH oxidase directly contributes to ROS production, both of which promote ferroptosis (Jiang et al., 2021). Doxorubicin treatment results in the suppression of GPX4, upregulation of PTGS2 (another key ferroptosis indicator) and lipid peroxidation of mitochondrial membrane, typical phenotypes of mitochondria-dependent ferroptosis, implying that anthracyclines induce ferroptosis in cardiomyocytes and hearts (Fang et al., 2019; Tadokoro et al., 2020; He et al., 2021). Consistently, inhibition of ferroptosis by pharmacological inhibitors (ferrostatin-1 or mitoTEMPO) or by genetic overexpression of GPX4 both significantly reduce doxorubicin-induced heart injury and mortality in mice (Fang et al., 2019; Tadokoro et al., 2020). Mechanistically, doxorubicin facilitates nuclear accumulation of the nuclear factor erythroid 2-related factor 2 (NRF2) and enables NRF2 to promote the expression of heme oxygenase 1 (HMOX1). HMOX1 can degrade heme to release free iron. As a result, iron accumulates in the heart after doxorubicin treatment, ultimately promoting mitochondrial-dependent ferroptosis (Fang et al., 2019). Moreover, anthracyclines downregulate GPX4, especially mitochondrial GPX4, thereby dampening the anti-ferroptosis effect of GPX4 and exacerbating cardiomyocyte injury (Tadokoro et al., 2020). Notably, inhibition of ferroptosis displays more efficient cardiac protection compared to repression of other types of programmed cell death, including apoptosis, necrosis and autophagy (Fang et al., 2019), further emphasizing the dominant role of iron-overload and ferroptosis in anthracycline-induced cardiotoxicity. Thus, ferroptosis inhibitors, such as ferrostatin-1 or mitoTEMPO, could be promising drugs to prevent anthracycline cardiotoxicity.

Overall, ferroptosis raises as a newly recognized programmed cell death. Past and recent evidence both highlight that anthracycline-induced iron-overload in mitochondria and the consequent mitochondria-dependent ferroptosis are dominant factors involved in anthracycline cardiotoxicity. It is worth further efforts to investigate whether ferroptosis inhibitors could act as cardioprotectant against anthracycline cardiotoxicity in the future.

## Autophagy, Mitophagy and Mitochondrial Dynamics in Anthracycline Cardiotoxicity

### Autophagy and Mitophagy in Anthracycline Cardiotoxicity

Autophagy is another programmed cell death process that has received great attention. Since its discovery, autophagy is considered as a beneficial process that helps to maintain cellular homeostasis by facilitating the removal of aggregated proteins and damaged organelles, such as mitochondria (Chun and Kim, 2018). Nevertheless, uncontrolled amplification of autophagy can also be a final outcome, resulting in cell death (Nishida et al., 2008). Autophagy starts with the nucleation and elongation of double membrane vesicles, named autophagosomes, which can encapsulate ubiquitinated proteins destined to be degraded (Russell et al., 2013). The recognition of cargo proteins or organelles depends on matured microtubule-associated protein 1 light chain 3 (LC3-II) on the autophagosome membranes and proteins that carry a LC3-II interacting domain (LIR), such as sequestosome-1 (SQSTM1, also known as ubiquitin-binding protein p62) (Klionsky et al., 2021). Consequently, autophagosomes fuse with lysosomes to form autolysosomes, wherein the low pH environment and proteases control the degradation of targeted protein cargos. Autophagy is strictly regulated by the upstream signalling, including AMP activated protein kinase (AMPK)-regulated activation and PI3K/Akt/mTOR-mediated inhibition (Kim et al., 2011).

The detoxifying effect of this cellular recycling process in other types of cardiac diseases is well recognized (Sabbah et al., 2016), but whether autophagy is beneficial or detrimental in anthracycline-induced cardiotoxicity is still a subject of dispute. Previous studies reported that anthracyclines stimulate cardiac autophagy, which primarily contributes to cardiomyocyte death after drug treatment. An enhanced level of autophagy markers, including Beclin1, LC3-II, p62, autophagy-related protein (ATG) 5 (ATG5) and 7 (ATG7), are detected in anthracycline-treated cardiomyocytes and hearts (Zhang et al., 2011, 2019; Wang et al., 2014; Luo et al., 2018). In support of these findings, inhibition of Beclin1 attenuates anthracycline-induced cardiomyocyte death, whereas Beclin1 overexpression exacerbates anthracycline cardiotoxicity (Kobayashi et al., 2010). However, other studies demonstrated that anthracyclines in fact can block autophagy and that promotion of cardiac autophagy flux is protective against anthracycline cardiotoxicity (Kawaguchi et al., 2012; Sishi et al., 2013; Dutta et al., 2014; Li et al., 2016, 2018; Song et al., 2018; He et al., 2021). These controversial findings may be attributed to a lack of uniform models to study the role of autophagy in anthracycline cardiotoxicity [reviewed in ref. (Li M. et al., 2020)]. Whether autophagy is protective or detrimental in anthracycline cardiotoxicity may be attribute to multiple reasons, including the lack of accurate analysis of autophagy flux, dose and duration of anthracycline treatment, as well as dynamic phases of the pathological development. Despite that no consensus has been made on the role of autophagy in anthracycline cardiotoxicity, most studies support the view that anthracyclines impair autophagy flux, resulting in the blockage of clearance and recycling of potentially toxic cellular components, such as damaged/dysfunctional mitochondria (Figure 1C).

Cardiomyocytes are abundant with mitochondria, which are the major source of ROS production. In turn, cardiomyocyte mitochondria are susceptible to ROS-induced damage. Mitochondrial autophagy, also called mitophagy, is essential for clearing damaged mitochondria and maintaining the functional ones (Saito and Junichi, 2015). In addition to the standard process of autophagy, mitophagy involves the participation of specific proteins, which work as labels for helping damaged-mitochondria recognition by LC3 and p62. These proteins include: 1) BCL2/adenovirus E1B 19 kDa interacting protein 3 (Bnip3), NIP3-like protein X (Nix, also known as Bnip3L), FUN14 domain containing 1 (FUNDC1) and cardiolipin, which are direct receptors of LC3 on the mitochondrial outer membrane, and 2) PTEN-inducible putative kinase 1 (PINK1) and Parkinson juvenile disease protein 2 (Parkin), which regulate mitochondrial protein ubiquitination and the recognition by p62 and LC3 (Saito and Junichi, 2015).

PINK1/Parkin-mediated recognition of damaged mitochondria by autophagosomes is the most studied mitophagy mechanism. PINK1 is a mitochondrial serine/threonine-protein kinase that locates in inner mitochondrial membrane. However, PINK1 fails to insert into the inner membrane when mitochondrial depolarization occurs, thereby accumulating at the outer mitochondrial membrane. Accumulated and activated PINK1 consequently recruits Parkin, an ubiquitin E3 ligase that ubiquitinates various proteins on the mitochondrial outer membrane, thus marking depolarized mitochondria to be recognized by p62, LC3 and autophagosomes (Narendra et al., 2008). Mitophagy is impaired in anthracycline-treated hearts, as a significant decrease of PINK1, Parkin and p62 in the mitochondria is detected after doxorubicin treatment (Hoshino et al., 2013). Precise analysis revealed that PINK1 and Parkin are suppressed by doxorubicin in early stages (2–8 days after treatment), but restore at later phases (14 days after treatment) (Hull et al., 2016). The mechanism about how anthracyclines inhibit PINK1, Parkin and the mitophagy process is still unclear, but may include the participation of apoptotic protein p53. Anthracyclines activate p53, subsequently promoting its direct binding and sequestering Parkin in the cytosol, thus hindering the recruitment of Parkin to mitochondria and preventing mitophagy initiation (Hoshino et al., 2013). Accordingly, both p53 deficiency and Parkin overexpression in cardiomyocytes restore mitophagy and attenuate anthracycline-induced cardiotoxicity (Hoshino et al., 2013).

Apart from the well-described PINK1/Parkin mechanism, Bnip3 and Nix (Bnip3L) are also essential players involved in mitophagy. Bnip3 and Nix are transcriptionally activated by hypoxia-HIF1α signalling axis and translocate to the outer mitochondrial membrane to trigger mitophagy. Notably, Bnip3 and Nix mediate mitophagy independent of the mitochondrial permeability transition pore opening and depolarization (Quinsay et al., 2010). Bnip3 contains a LIR domain that allows it to directly interact with LC3 and guide the recognition of mitochondria by autophagosome (Zhang and Ney, 2009; Saito and Junichi, 2015). Anthracyclines upregulate Bnip3 expression in a dose-dependent manner and promote its translocation to mitochondria (Dhingra et al., 2014). In line with a maladaptive role of Bnip3 in anthracycline cardiotoxicity, inhibition of Bnip3 with shRNA and expression of a mutant Bnip3 that lacks a mitochondria-binding domain, both rescued anthracycline-induced mitochondrial dysfunction in cardiomyocytes. Bnip3 deficiency (Bnip3^−/−^) also protected the mice against anthracycline-induced mitochondrial injury and improved their survival (Dhingra et al., 2014). It is still unclear how anthracyclines activate Bnip3 in the heart. Anthracycline-induced ROS could be a trigger, as it is found that oxidative stress induces Bnip3 dimerization and activation in ischemia/reperfusion (I/R) hearts (Kubli et al., 2008). Notably, Bnip3 regulates both necrosis and the mitophagy process. It is worthy to further clarify whether both mechanisms are involved in Bnip3-mediated heart injury after anthracycline treatment.

Similar to the debate in autophagy, the role of mitophagy in anthracycline-induced cardiotoxicity is also unclear. Current evidence supports the view that anthracyclines inhibit PINK1/Parkin-regulated mitophagy and that restoration of mitophagy rescues anthracycline cardiotoxicity. In contrast, Bnip3, a dual marker of mitophagy and necrosis is upregulated by anthracyclines, prior to mitochondrial polarization. Anthracyclines appear to induce Bnip3 activation and promote necrosis (Dhingra et al., 2014), but it still needs to be clarified whether Bnip3-regulated mitophagy is involved in anthracycline cardiotoxicity. Moreover, chronic low-dose (Hoshino et al., 2013; Hull et al., 2016) and acute high-dose (Dhingra et al., 2014) anthracycline treatment regimens are used in different studies, resulting in the difficulty to reach a consensus whether mitophagy is injurious or protective during anthracycline cardiotoxicity.

### Mitochondrial Dynamics in Anthracycline Cardiotoxicity

Mitochondria are highly dynamic organelles that change their morphology through fission and fusion. This process, namely mitochondrial dynamics, helps mitochondria to adapt to cellular stresses such as energy deficiency and ROS-induced injury, thereby ensuring mitochondrial function (Youle and van der Bliek, 2012). The fission process generates small spherical mitochondria, while the fusion process produces extended mitochondria and mitochondrial networks. Mitochondrial dynamics and autophagy/mitophagy constantly work together to eliminate injured and dysfunctional mitochondria. High resolution structured illumination microscopy (SIM) clearly showed that smaller mitochondria derived from mitochondria fission are always accompanied with a higher level of ROS and loss of membrane potential, compared to the larger fission portion. These smaller mitochondria are destined for being degraded by mitophagy (Kleele et al., 2021). The mitochondrial fission process is mediated by fission factors, including mitochondrial fission factor (MFF), mitochondrial fission process 1 (MTFP1), dynamin-related protein 1 (DRP1), mitochondrial dynamics proteins of 49 kDa [MiD49 (also known as MIEF2)] and 51 kDa [MiD51 (also known as MIEF1)] (Kraus et al., 2021), whereas mitochondrial fusion is regulated by specific GTPases, including mitofusin 1 (MFN1), mitofusin 2 (MFN2) and optic atrophy 1 (OPA1) (Tilokani et al., 2018).

Dysregulation of mitochondrial dynamics were observed in various heart diseases, including cardiac hypertrophy, heart failure, dilated cardiomyopathy (DCM), ischemic heart disease [reviewed in ref (Ikeda et al., 2014)], as well as anthracycline-induced cardiomyopathy [reviewed in ref (Osataphan et al., 2020)]. Doxorubicin can decrease the expression of MFN1, MFN2 and OPA1, but increase DRP1 and promote mitochondria fragmentation (Li et al., 2014; Tang et al., 2017; Marques-Aleixo et al., 2018b; Catanzaro et al., 2019), suggesting that anthracyclines may inhibit mitochondrial fusion and promote mitochondrial fission. In rodent cardiomyocytes and hearts, doxorubicin exposure promotes phosphorylation of DRP1 at serine 616, which is essential for the activity of DRP1 to mediate mitochondrial fission (Dhingra et al., 2017; Xia et al., 2017). In line with this, DRP1 deficient animals are protected against anthracycline-induced cardiac dysfunction (Catanzaro et al., 2019). In addition, mitophagy inhibitor liensinine mitigates anthracycline-induced cardiotoxicity by inhibiting DRP1-mediated mitochondrial fission (Liang et al., 2020). These findings together suggest that anthracycline cardiotoxicity is mediated, at least in part, by the increased mitochondrial fragmentation, fission and the accelerated mitochondrial degradation by mitophagy. Inhibiting mitochondrial fission and mitophagy may provide beneficial effect against anthracycline cardiotoxicity. However, further studies are required to confirm whether the change of mitochondrial dynamics is a consequent of anthracycline-induced mitochondrial damage or a direct driver of cardiac injury.

## Anthracycline-Induced Cardiac Metabolic Disturbance

As discussed above, mitochondria are the key energy factories of cardiomyocytes and major targets of anthracyclines. It is thus no doubt that anthracycline-induced long-term mitochondrial dysfunction can result in energy deficiency in cardiomyocytes. In fact, we and others all revealed that ATP production is impaired by anthracyclines (Li et al., 2018; Abdullah et al., 2019). Mitochondria isolated from acute and chronic anthracycline-treated hearts both showed significant suppression of mitochondrial respiration (Abdullah et al., 2019). Therefore, metabolic disturbance may also be a mechanism underlying anthracycline-induced cardiomyopathy (Figure 1D).

### Anthracyclines Interrupt Myocardial Substrate Utilization

In physiological condition, hearts mainly use fatty acids (FAs) as substrates and rely on TCA cycle for ATP production. However, in the failing hearts, where mitochondria are impaired by ROS or other insults, cardiomyocytes switch to use glucose, lactate and a small amount of ketone bodies for energy production. Glycolysis then raises as a substituted metabolic mode to produce ATP when OXPHOS is suppressed (Doenst et al., 2013). In fact, anthracyclines increase translocation of glucose transporter 1 (GLUT1) to the plasma membrane (Hrelia et al., 2002), thereby enhancing transient glucose uptake in isolated cardiomyocytes (Hrelia et al., 2002), as well as in hearts of mice and Hodgkin disease patients (Bauckneht et al., 2017; Sarocchi et al., 2018). In line with this, an increase of glycolytic flux (Carvalho et al., 2010) and key glycolytic enzymes, such as hexokinase (HK), phosphofructokinase (PFK) and pyruvate kinase (PK) (Li et al., 2018), were found in anthracycline-treated hearts. Nevertheless, glycolysis activation seems to be a transient and compensatory response that cannot meet the cellular energy demand in the long term, as glucose uptake returns to basal level later (3 h) after anthracycline treatment in cardiomyocytes (Hrelia et al., 2002). Moreover, persistent glucose usage may lead to several glucose-dependent non-ATP-generating pathways, such as pentose phosphate pathway (PPP), which serves as an important source of NADPH and ROS, and hexosamine biosynthetic pathway (HBP), which regulates protein O-GlcNAcylation (Doenst et al., 2013).

In contrast, long-chain fatty acid oxidation is inhibited in anthracycline-treated rat hearts (Carvalho et al., 2010). For instance, palmitate oxidation was reduced by 40–70% in cardiomyocytes isolated from acute or chronic anthracycline-treated animals (Abdel-aleem et al., 1997; Sayed-ahmed et al., 1999). Mechanistically, anthracyclines interrupt the activity of carnitine palmitoyl transferase I (CPT I), a mitochondrial membrane enzyme that converts long-chain acyl-CoA to long-chain acyl-carnitines and facilitates their transport into mitochondria. Therefore, anthracyclines inhibit the fatty-acid ß-oxidation process (Abdel-aleem et al., 1997). Accordingly, supplementation of L-carnitine was proved to attenuate anthracycline cardiotoxicity in animals (Andrieu-Abadie et al., 1999; Sayed-ahmed et al., 2000; Cabral et al., 2018) and in patients with non-Hodgkin lymphoma (Waldner et al., 2006).

Overall, when anthracyclines induce mitochondrial dysfunction, glucose oxidation is transiently activated while fatty acid oxidation is inhibited. The alteration of substrate utilization compensates early energy loss, but cannot meet the long-term cellular energy demand, thus resulting in maladaptive energetic failure. Strategies aiming for supplementing additional ATP production may work to mitigate anthracycline cardiotoxicity. For instance, an early study demonstrated that rats continuously administrated with fructose-1,6-diphosphate, a glycolytic intermediate, displayed significant improvement of cardiac function after acute and chronic anthracycline treatment (Danesi et al., 1990). Stimulating mitochondrial respiration, by L-carnitine supplementation (Andrieu-Abadie et al., 1999; Sayed-ahmed et al., 2000; Cabral et al., 2018) or with OXPHOS medium filled with galactose/pyruvate/glutamine (Deus et al., 2015), also restored myocardial energy supply and mitigated anthracycline cardiotoxicity.

### AMPK-Mediated Metabolic Homeostasis During Anthracycline Cardiotoxicity

AMPK is the main intracellular sensor and regulator of energy and nutrient signalling. It is canonically activated by the increase of AMP:ATP or ADP:ATP ratios that result from a variety of energy stresses, including starvation, exercise, ischemia or mitochondrial dysfunction. AMP and ADP can directly bind AMPK and promote its phosphorylation by liver-kinase-B1 (LBK1), thus increasing AMPK activity up to 100-fold. In contrast, ATP inhibits AMPK phosphorylation and activation. In addition, AMPK can also be phosphorylated by calcium/calmodulin-dependent kinase kinase 2 (CAMKKß) that is activated by different hormone stimulations. Once activated, AMPK can phosphorylate and regulate a variety of downstream proteins to promote catabolic processes such as fatty acid oxidation, glucose uptake, glycolysis, autophagy and mitochondrial fission, or inhibit anabolic processes such as synthesis of protein, fatty acid, glycogen and sterol (Garcia and Shaw, 2017).

Despite that anthracyclines induce mitochondrial dysfunction and energy deficiency, which are supposed to stimulate AMPK signalling, many studies revealed that AMPK as well as its downstream target acetyl-CoA carboxylase (ACC) are indeed inhibited by anthracyclines (Gratia et al., 2012; Kawaguchi et al., 2012; Wang et al., 2012). Accordingly, AMPKα1 deficient (AMPKα1^−/−^) mouse embryonic fibroblasts and cardiomyocytes are more susceptible to anthracycline treatment (Wang et al., 2012), but AMPK activation by a variety of strategies, such as prior starvation, 5-aminoimidazole-4-carboxamide ribonucleoside (AICAR) treatment and adenovirus-mediated AMPK constitutive activation (AMPK-CA), diminishes anthracycline-induced heart injury (Chen et al., 2011; Kawaguchi et al., 2012; Wang et al., 2012). In this view, instead of serving as a trigger to activate AMPK, energetic failure seems to be a result of anthracycline-regulated AMPK inhibition. As a result, catabolic processes aiming for increasing ATP production under anthracycline stress are inhibited following AMPK inhibition. This partially explains the inhibition of autophagy by anthracyclines, as AMPK inhibition results in direct reduction of ULK1 phosphorylation and blockage of autophagy initiation (Chen et al., 2011; Kawaguchi et al., 2012). Moreover, AMPK inhibition also reactivates mTORC1, which further promotes energy consumption, inhibits autophagy and worsens energetic stress (Gratia et al., 2012). Metformin, a well-recognized AMPK activator (Hawley et al., 2002), has also showed promising protective effects against anthracycline-induced heart injury (Asensio-López et al., 2011; Kobashigawa et al., 2014; Zilinyi et al., 2018). This further supports a fundamental role of AMPK in anthracycline-mediated metabolic disturbance. Nevertheless, the mechanism underlying how anthracyclines inhibit AMPK is still unclear and awaits further investigation.

Taken together, AMPK signalling is inhibited by anthracyclines, leading to the exacerbation of cardiac energetic stress, which further contributes to anthracycline cardiotoxicity. Reactivation of AMPK could be a potential strategy to attenuate anthracycline cardiotoxicity.

## Strategies Targeting Mitochondrial Mechanism to Reduce Anthracycline Cardiotoxicity

Mitochondria are crucial for anthracycline-induced cardiotoxicity (Figure 1). Targeting mitochondrial mechanisms could provide potential strategies to attenuate anthracycline cardiotoxicity (Murabito et al., 2020). Anthracyclines promote excessive ROS production within the cytoplasm and mitochondria. Thus, antioxidants are initially tested in early studies to counterwork anthracycline cardiotoxicity. Nevertheless, antioxidants or ROS scavengers, such as the xanthine oxidase inhibitor allopurinol, the NADPH oxidase inhibitors apocynin and DPI, coenzyme Q10, L-carnitine, NAC and vitamins E and C, failed to mitigate anthracycline cardiotoxicity in animal (Berthiaume et al., 2005; Mukhopadhyay et al., 2009) and clinical studies (van Dalen et al., 2011), suggesting that general inhibition of ROS production is not sufficient to prevent anthracycline-induced cardiotoxicity. Moreover, some antioxidants are able to decrease anthracycline-induced oxidative stress but without preventing mitochondrial dysfunction (Berthiaume et al., 2005), implying that specifically targeting mitochondria-related ROS generation may be requisite. MitoTEMPO, a derivative of TEMPO, is a mitochondria-targeted superoxide mimetic. It can readily pass through lipid bilayers, accumulate in mitochondria, enhance superoxide dismutase (SOD) activity and scavenge mitochondrial O_2_^−^. Studies revealed that mitoTEMPO, but not TEMPO, can suppress anthracycline-induced lipid peroxidation specifically in mitochondria and reduce cardiomyopathy (Rocha et al., 2016; Fang et al., 2019). Other mitochondria-targeted antioxidants, like mitoquinone (mitoQ), also display protective effect against anthracycline-induced cardiotoxicity and endotheliotoxicity (Chandran et al., 2009; Clayton et al., 2020; Wu et al., 2020). Thus, mitoTEMPO and mitoQ are promising drugs to mitigate anthracycline-induced cardiomyopathy.

As discussed above, iron accumulates in mitochondria during anthracycline exposure. Therefore, iron chelators were primarily considered as pharmacological strategies to limit anthracycline cardiotoxicity. Nevertheless, dexrazoxane currently represents the only iron-chelating drug approved by FDA and EMA to prevent anthracycline-induced cardiomyopathy in breast cancer patients. The protective effect of dexrazoxane is primarily attributed to the scavenging of free iron (Xu et al., 2005). However, other iron chelators, such as desferrioxamine and deferasirox, failed to achieve the same protective effect (Voest et al., 1994; Hasinoff et al., 2003), suggesting that dexrazoxane may protect the heart against anthracycline cardiotoxicity through mechanisms more than iron chelating. Later works revealed that dexrazoxane can also target and inhibit topoisomerase IIß (TOP2ß), avoiding the formation of anthracycline-TOP2ß-DNA cleavage complex and the consequent DNA double-strand breaks (Lyu et al., 2007; Hasinoff et al., 2020). The cardiac protective effect of dexrazoxane was proved in animal models (Alderton et al., 1990; Bjelogrlic et al., 2007) and was further validated in clinical trials on anthracycline-treated breast cancer patients (Venturini et al., 1996; Swain et al., 1997; Lopez et al., 1998). Nonetheless, its clinical use was initially restricted by FDA and EMA to adult breast cancer patients who receive therapeutic dose higher than 300 mg/m^2^ doxorubicin or 540 mg/m^2^ epirubicin, since it was debated that dexrazoxane may induce secondary hematological malignancies in pediatric patients (Tebbi et al., 2007; Lipshultz et al., 2010). However, new studies based on a large patient collection stated that dexrazoxane is cardioprotective and safe in pediatric leukemia patients receiving anthracycline therapy (Asselin et al., 2016). The EMA recently removed this contraindication for children and adolescents treated with high cumulative doses of anthracyclines (European Medicines Agency, 2018b).

Ferroptosis is a newly recognized programmed cell death that relies on both ROS generation and iron homeostasis disruption (Jiang et al., 2021). Studies in recent years highlighted that ferroptosis could be the dominant programmed cell death induced by anthracyclines, as inhibition of ferroptosis shows more efficient cardiac protection compared to blockage of apoptosis, necrosis or autophagy (Fang et al., 2019). Ferrostatin-1 is a widely-used ferroptosis inhibitor, potentially functioning through scavenging the alkoxyl radicals, interacting with ferrous iron and inhibiting lipid peroxidation (Miotto et al., 2020). Many studies have demonstrated that ferrostatin-1 efficiently attenuates anthracycline-induced ferroptosis, mitochondrial dysfunction and heart injury (Fang et al., 2019; Tadokoro et al., 2020; He et al., 2021). Notably, although ferrostatin-1 and dexrazoxane both inhibit ferroptosis and show similar protection against anthracycline-induced cardiac damage, they seem to function through different mechanisms. Ferrostatin-1 does not reduce the labile iron pool in the heart, whereas dexrazoxane attenuates cardiac free irons, after anthracycline treatment (Fang et al., 2019). Moreover, concomitant inhibition of ferroptosis and apoptosis with ferrostatin-1 and zVAD-FMK is more efficient than treating with ferrostatin-1 or Zvad-FMK alone and fully prevents anthracycline-induced cardiomyocyte death (Tadokoro et al., 2020). This suggests that anthracycline-induced cell death is not a single-factorial event.

The roles of autophagy and mitophagy in anthracycline-induced cardiotoxicity have been studied for decades, however, there is currently no autophagy-targeting compound being used in preclinical trials to reduce anthracycline cardiotoxicity. This is largely due to the controversy whether autophagy/mitophagy is beneficial or detrimental for anthracycline cardiotoxicity (Li M. et al., 2020). Nevertheless, some attempts targeting autophagy/mitophagy have been tried in animal models. Autophagy activators, such as mTOR inhibitor (rapamycin) (Ding et al., 2011), PI3Kγ inhibitors (AS605240, IPI145) (Li et al., 2018), show protective effect against anthracycline-induced heart injury in zebrafish and mouse. Other nonpharmacological interventions to promote autophagy, including prior starvation (Kawaguchi et al., 2012) and caloric restriction (Chen et al., 2011), also display cardioprotection during anthracycline treatment. Some studies suggested that inhibition of autophagy initiation could be beneficial, as anthracyclines inhibit the lysosome acidification, resulting in an accumulation of autophagosomes and autolysosomes (Li et al., 2016). In this regard, promotion of autophagy flux could be more precise than simple activation or inhibition of autophagy to prevent anthracycline cardiotoxicity. Unfortunately, selective drugs that target lysosome/autolysosome and facilitate autophagy flux are still not available. In contrast, inhibition of mitochondrial fission and mitophagy seems to be beneficial. The mitophagy inhibitor liensinine protects the heart against anthracycline-induced ROS generation and contractile dysfunction by decreasing DRP1 phosphorylation, inhibiting mitochondrial fragmentation and reducing mitophagy (Liang et al., 2020).

Mitochondria are the well-known energy factories of cardiomyocytes. Thus, anthracycline-induced mitochondrial dysfunction can ultimately lead to energy deficiency and metabolic disturbance. Strategies aiming at improving mitochondrial function could be capable to mitigate anthracycline cardiotoxicity. For example, elamipretide is a tetrapeptide designed to selectively target mitochondrial ETC and restore cellular bioenergetics. Animal studies (Gupta et al., 2016; Sabbah et al., 2016) and a small clinical trial (Daubert et al., 2017) all revealed that elamipretide can improve mitochondrial function and preserve cardiac contractility in canines and patients with heart failure. Although there is no direct evidence proving that elamipretide can prevent anthracycline-induced cardiomyopathy, the common feature of mitochondrial dysfunction during anthracycline cardiotoxicity suggests that elamipretide could be a promising drug. Interestingly, a recent work reported a mitochondria supplementation strategy in which mitochondria-rich extracellular vesicles (EVs), isolated from mesenchymal stem cells (MSCs), were transported to patient pluripotent stem cell-derived cardiomyocytes (iCMs). Mitochondria-rich EVs significantly promote ATP production and mitochondrial biogenesis and rescue iCMs from doxorubicin injury (O’Brien et al., 2021; Wang et al., 2021). Further, a recent work overviewed the tolerability and efficacy of exercise on cancer therapy-induced cardiovascular toxicity and concluded that aerobic exercise can improve peak oxygen uptake (VO2peak) and cardiorespiratory fitness (CRF) by an integrative assessment of global cardiovascular function (Scott et al., 2018). Physical activity was proved to mitigate anthracycline-induced mitochondriopathy in multiple animal studies [reviewed in ref (Marques-Aleixo et al., 2018a)]. The beneficial effect of exercise training primarily stems from the activation of mitochondrial biogenesis and adaption (MacInnis and Gibala, 2017), as well as the stimulation of energetic pathways such as AMPK signalling and the autophagy process (Marques-Aleixo et al., 2018b). Together, these animal and clinical evidence suggest that exercise training could be another potential strategy to prevent cardiotoxicity induced by anti-cancer therapy.

AMPK is a central mediator for cardiac metabolic homeostasis. A large set of studies proved that AMPK activators, like metformin (Asensio-López et al., 2011; Kobashigawa et al., 2014; Zilinyi et al., 2018) and melatonin (Liu et al., 2018; Li H. et al., 2020; Najafi et al., 2020; Arinno et al., 2021), can efficiently alleviate anthracycline-induced cardiac injury. The mechanism underlying the protective effect of AMPK activators is multi-factorial, for example, AMPK can facilitate fatty acid oxidation and promote cardiac autophagy. Based on these studies, a phase II clinical trial were carried out to validate whether metformin could reduce doxorubicin-induced cardiotoxicity in breast cancer patients (Avera McKennan Hospital & University Health Center, 2019). Unfortunately, this clinical trial was terminated due to slow accrual. Future clinical trials are needed to answer whether metformin or other AMPK activators could prevent anthracycline cardiotoxicity.

## Conclusion

Cardiotoxicity induced by anthracyclines remains a hard stone that hampers their clinical applications in chemotherapy. Despite that the molecular mechanism underlying anthracycline cardiotoxicity is complex and multifactorial, emerging evidence supports the ideas that cardiac mitochondria are one of the pivotal targets of anthracyclines and mitochondrial dysfunction plays a determinant role in anthracycline cardiotoxicity (Figure 1). Anthracyclines have high affinity to the inner mitochondrial membrane protein cardiolipin and thus preferentially accumulate in mitochondria. Moreover, the special structures of anthracyclines decide that they are ideal substrates for redox reaction that produces excessive ROS which in turn injures mitochondria. In addition, anthracyclines interrupt iron metabolism and induce labile iron overload in mitochondria, which lead to cellular toxicity along with surplus ROS production. Thus, iron-dependent ferroptosis arises as a dominant programmed cell death that attributes to anthracycline-induced cardiomyocyte loss. Conversely, flux of another programmed cell death, autophagy, is impaired by anthracyclines. This restrains the efficient degradation and recycling of damaged cellular components including mitochondria, thereby exacerbating anthracycline-induced injury. Furthermore, anthracycline-associated mitochondrial dysfunction may reprogram the utilization of fuel substrates, including glucose and fatty acids, ultimately inducing metabolic disturbance and energetic failure.

Based on these aspects, pharmacological strategies aiming at reducing mitochondrial injury or restoring mitochondrial function could potentially lessen anthracycline cardiotoxicity. These strategies include the utilization of mitochondrial specific antioxidants, iron chelators, ferroptosis inhibitors, autophagy/mitophagy compounds and mitochondrial energetic stimulators. Some of these drugs showed protective effects in cardiomyocytes *in vitro* or animal models *in vivo*, yet failed to provide further benefits in preclinical studies. Despite almost 50 years of research, dexrazoxane remains the only FDA- and EMA-approved protectant to reduce cardiotoxicity in adult breast cancer patients who receive accumulative high dose anthracycline therapy. This could be due to the fact that anthracycline cardiotoxicity is a multi-factor event. For instance: 1) Dexrazoxane succeeds because it functions both as an iron chelator to reduce iron-overload and a TOP2ß inhibitor to mitigate DNA cleavage. 2) Antioxidant or iron chelator alone shows limited effects against anthracycline cardiotoxicity, while inhibition of ferroptosis by scavenging ROS and limiting iron-overload together displays more significant protection. 3) Concomitant inhibition of ferroptosis and apoptosis is more efficacious than supressing each process alone. The joint regimen fully prevents anthracycline-induced cardiomyocyte death. In the future, more bench work, preclinical and clinical studies are worth further testing, on one hand, whether the aforementioned mitochondria-targeted drugs could prevent anthracycline-induced cardiotoxicity and, on the other hand, whether targeting different mechanisms together could provide more efficient cardiac protection.
